# Sap flow of sweet cherry reveals distinct effects of humidity and wind under rain covered and netted protected cropping systems

**DOI:** 10.1038/s41598-022-25207-0

**Published:** 2022-12-05

**Authors:** C. H. Stone, D. C. Close, R. Corkrey, I. Goodwin

**Affiliations:** 1 Tasmanian Institute of Agriculture, University of Tasmania, Hobart, 7001 Australia; 2 Agriculture Victoria, Tatura, Australia; 3 Centre for Agricultural Innovation, The University of Melbourne, Melbourne, Australia

**Keywords:** Plant sciences, Agroecology, Ecophysiology

## Abstract

Protected cropping systems (PCS) alter the plant growing environment, though understanding of this in ventilated systems and how the new climate affects tree water uptake is limited. Sap flow sensors and weather stations were deployed in 16-year-old ‘Lapins’ on ‘Colt’ rootstock cherry trees under a ventilated Voen PCS and in an adjacent bird netted PCS. Average and maximum temperatures were consistently higher (14.7 °C and 22.9 °C) while total daily solar radiation and average wind were consistently lower (12.9 MJ/m^2^ and 0.2 m/s) in rain covered, in contrast to netted, PCS (13.9 °C, 21.3 °C, 13.7 MJ/m^2^ and 0.9 m/s). Over the season, a threefold lower daily sap flow rate was observed under rain covered PCS. Using generalised additive modelling (GAM), the influence of individual climate parameters on sap flow were predicted. Whilst sap flow was only slightly affected by relative humidity (RH) less than 60%, above this threshold sap flow rapidly declined under rain covered PCS whereas sap flow more gradually declined above 20% RH under netted PCS. Overall, our novel modelling approach led to the discovery of the 60% RH critical threshold on predicted sap flow and the indirect effect that wind speeds have on sap flow under PCS.

## Introduction

A protective cropping system (PCS) is the term commonly used to define a structure either permanent or temporary that provides shelter to a valuable commodity from environmental conditions^1^. In its broadest definition this includes the use of glasshouses and greenhouses, shade houses, rain covers and bird exclusion netting. The use of bird exclusion netting is standard practice in sweet cherry production. Rain covers are becoming more prevalent in cherry orchards primarily to counter the risk of late season rainfall events^2–4^. Research focussing on tree water use (via sap flow techniques) under PCS has predominantly been undertaken in glasshouse environments with young trees growing in bags or within orchards under net^5–8^. It has been reported that PCS change the mesoclimate of an orchard, exposing the trees and fruit to warmer air and soil temperatures, lower vapour pressure deficits (VPD) and a reduction in wind and incident levels of photosynthetic active radiation (PAR)^9^, all of which can impact on rates of tree transpiration. Passive PCS (high tunnels) have been associated with high temperature and humidity levels and filtered solar radiation, with negative impacts on fruit quality characteristics of sweet cherry^10–12^. New rain covered PCS have been developed that facilitate greater levels of venting. However, there is limited research on the mesoclimate under modern rain covered PCS and how these impact tree water use, and hence irrigation and fertigation needs.

Bird and hail exclusion netting over orchards has been found to improve water use efficiency. Nicolás et al.^7^ found higher leaf stomatal conductance and photosynthetic rates in netted apricot trees compared to exposed trees, that was associated with a 10–20% reduction in daily sap flow. Similarly, leaf conductance of citrus trees increased under dense netting (4.1 mm/s) compared to sparse netting (2.9 mm/s) and a no netting control (1.8 mm/s) while daily total sap flows were reduced under dense netting by 6–7% with no significant change under sparse netting. The average reduction in midday solar radiation was 53% and 27% for dense and sparse netting respectively with cooler midday sunlit (exposed) leaf temperatures measured under the netting systems^13^.

Whilst the effects of elevated temperatures and VPD on photosynthesis and sap flow rates in trees under PCS have been published^14–16^, to our knowledge the effects of RH and wind on sap flow under PCS in field conditions have not been reported. Laboratory trials on the effects of wind^17^, CO_2_ and temperature^18,19^ on sap flow and stomatal conductance have been reported, with Urban et al.^19^ finding that stomatal conductance increased with rising temperatures (up to 40 °C) when VPD was held constant at 1 kPa despite a decrease in leaf water potential. This direct stomatal response to increasing temperatures improved the evaporative cooling during periods of high heat as well as enabling the benefit of less stomatal limitation to photosynthesis^19^. In addition to leaf shape, Laplace et al.^17^ concluded that stomatal conductance could be a dominant factor affecting how wind speeds affect rates of transpiration across plant species. Enriched CO_2_ environments reduced tree sap flow rates in 30-year-old Scots pine (*Pinus sylvestris*) by 14.4%, however elevated temperatures led to significantly higher sap flow rates (32.5%). There were no significant interactive effects on sap flow rates for trees exposed to both elevated temperatures and elevated CO_2_ levels, suggesting that temperature plays a dominant role on sap flow^18^. Finally, it has been reported that stem-stored water can provide for sap flow following over-night re-filling^20,21^. This phenomenon could potentially affect the influence of RH on sap flow during the hours of early morning such that there could be a time-lag between sap flow and effects of RH.

Of the various approaches to measurement of sap flow, the compensation heat-pulse method (CHPM)^22,23^ has been successfully used in many studies including on apple^24^, pear^25^ and olive^26^. In this study we have used the CHPM to investigate how sweet cherry tree water use responds to climatic variables, as influenced by rain covered and netted PCS, throughout the season. The aims of this study were to investigate, using a modelling approach the mesoclimate created by rain covered and netted PCS as well as to elucidate how the altered mesoclimates affect tree water use.

## Materials and methods

### Site characteristics

Field research was undertaken during the 2019–2020 growing season on a commercially managed sweet cherry orchard at Grove in the Huon Valley, Tasmania (43° 00ʹ S, 147° 05ʹ E). The region is classified as a cool temperate climate with a mean annual rainfall of 718 mm and mean temperatures of 14 °C during the growing months of December–February.

Two PCS blocks were utilised for this study: a ‘rain covered’ block (2 ha) planted on a north-eastern slope (9%) at 1.75 m within and 4.25 m between row spacings and a row length of 150 m, and an adjacent bird-exclusion ‘netted’ block (0.5 ha) on a north-easterly slope (6%), at 2 m within and 4.25 m between row spacings with a row length of 50 m. Soil profiles for both blocks comprised of a duplex soil with a loam topsoil over a heavy clay subsoil. Whilst soil moisture data was not available, irrigation and fertigation were applied (4 L h^−1^) through the same drip lines for both blocks which were situated immediately adjacent on the same soil type and standard grower practice was applied for all pest and weed management. Collection and analysis of all plant material in this study complies with institutional, national, and international guidelines and legislation.

### Rain covered PCS

Voen rain covers (Vöhringer GmbH & Co, Berg, Germany), made from polyethylene plastic reinforced with vented coverings (thickness 6 mm; 82% light transmission) sown into a hail net base were deployed over the rain covered block. These rain covers are designed to enhance ventilation while still protecting the tree canopies from direct rainfall, as well as being able to transmit adequate light levels for canopy and fruit development. The rain covers were deployed approximately 20 days after full bloom (DAFB) in spring on the 10^th^ of November for the rain covered block and remained in place until the 30th of March at the completion of the season. The netted block consisted of black bird-exclusion netting with a weave diameter of 2 cm.

### Trial design

16-year-old ‘Lapins’ on ‘Colt’ rootstock trained to a Spanish bush system were selected for this trial. ‘Colt’ is still the most widely used rootstock in Australia due to nursery availability and was certainly best practice when the trees used in this study were established. Two trees in every fourth row of the rain covered block were randomly selected and tagged (12 trees in total), while three trees in every third row were randomly selected and tagged in the netted block (9 trees in total). A buffer zone of approximately 12 m from the ends of the blocks was imposed to avoid edge effects. Trunk circumferences were measured 10 cm above the graft union and trunk cross-sectional areas (TCSA) calculated for each tree. Each tagged tree had three branches selected and tagged for flower and fruit set counts. Fruit from tagged branches were harvested for quality analysis at the end of the season.

### Sap flow

Two sets of heat-pulse probes (Tranzflo NZ Ltd, Palmerston North, New Zealand) were inserted in the tree trunks (north and west aspects) with a total of nine trees being measured (six under rain covers, three under net). A set of heat-pulse probes consisted of two 1.85 mm diameter temperature probes and a 1.85 mm diameter heater probe. Temperature probes were made from 19-g stainless hypodermic tube, with four thermocouples at depths of 5, 15, 25 & 40 mm insulated with a Teflon sheath. Using a steel guide and a 1.95 mm drill bit, three vertically aligned holes were drilled into the trunk. A corer was used to remove the bark at the probe sites, ensuring the 5 mm thermocouple would be situated as close to the cambium as possible. The probes were inserted between 9 and 29 cm above the ground with the standard spacing of 5 mm upstream and 10 mm downstream from the heater probe. Probes and the surrounding tree trunk were insulated from direct thermal radiation with aluminium foil. Sensors remained in the trees for nine months until May. The standard compensation heat-pulse method (CHPM) was used to calculate daily transpiration rates as per Green and Clothier^27^ and Green et al.^28^ from sap flux velocities > 2 cm/h. Data loggers (model CR1000, Campbell Scientific, Logan, USA) were used to measure the time taken to achieve thermal equilibrium between sensors located above and below the heater following the application of a 2.5 s heat pulse into the conducting wood area of the tree. If thermal equilibrium was not reached within a 480 s period, the sap flux density was set to 0. The average tree trunk circumferences were 48 cm (± 1.1) under bird netting relative to 40 cm (± 3) in the rain covered block. Thus for each tree, sap flux velocity (L/h) was calculated using the sapwood area (cm^2^) at each depth (5, 15, 25 and 40 mm). The sum of all four depths gives the volume flow per unit time per tree. The heat pulse for each probe set was regulated as per Green et al.^28^ to ensure that the heater probes delivered the same amount of energy (50 J) each time they were fired. Data were collected at 30-min intervals. Conducting wood area was calculated after taking core samples from representative trees. Daily sap flow was calculated using the approach outlined by Green et al.^24,29^, with calculations accounting for the wounding effect. Using the theoretical calibrations of Swanson and Whitfield^23^ a wound diameter of 2.8 mm was determined for the 1.98 mm diameter drill holes.

### Climate

Climate data was collected using Hobo U30-NRC weather stations (Onset, USA), which were positioned centrally in each of the two orchard blocks at canopy height (1.8 m), with a third station located in an open area between the two trial blocks. Each weather station measured air temperature (°C), relative humidity (RH) (%), global solar radiation (W/m^2^) and wind speeds (m/s) every 30 min. Wind speed measurements were averaged over the 30-min. Climate data was collected from October 2019 to June 2020. This data was used to estimate the respective hourly and daily values of vapour pressure deficit (VPD) and reference crop evapotranspiration (ETo). Crop factors were used to estimate crop evapotranspiration from ETo for each block (FAO-56; Allen et al.^30^).

## Data analysis

### Sap flow

Daily sap flow (24 h) was modelled using a statistical approach in which candidate parameters considered were daily average temperature (°C), maximum temperature (°C), daily solar radiation (MJ/m^2^), daily average relative humidity (%), minimum relative humidity (%) and wind speed (m/s). The analysis proceeded in three stages. The first was to model the change in sap flow trend in time-only using a nonlinear approach that accounted for repeated measures correlations and for the cyclical variation within each day. The distribution of the data was approximated using a bell shape curve of the form

1 $$SF= B \times esp \left(-\frac{{\left(\mathrm{Halhour}-\mathrm{M}\right)}^{2}}{S}\right).$$

This nonlinear model (Eq. 1) has a parameter ‘B’ that controls the height of the fitted curve; ‘M’ controls the time-position of its peak; and ‘S’ controls the width of the peak. This model included a Gaussian random effect for the `B' parameter for each day. The time-only model was fit using PROC NLMIXED in SAS version 9.4. Having obtained a good fit, the residuals versus time (30 min intervals) from the nonlinear model were then examined using a graphical method using R version 3.6 statistical software^31^.

In the second step the generalised additive models (GAM)^32^ were used to examine the deviations found in the time intervals. GAM models were fitted to the residuals from the time-only model using temperature, solar radiation, RH and wind speed as predictors. This allowed the effects of temperature, solar radiation, RH and wind speed on the mean estimate of maximum sap flow to be visually assessed within a non-parametric framework after having already allowed for the bell-shaped time-variation. Additional analysis was conducted to determine the possibility of a time lag of effects of RH on sap flow, due to stem-storage of water after overnight re-filling that might have consequences for modelled sap flow^20,21^. The nonlinear model was refitted using time lags of 0 (no lag), 1 (30 min), 2 (60 min) and 3 (90 min) to determine how well each described the data using Akaike information criterion (AIC)^33^. The GAMs were intended to serve an exploratory purpose rather than an inferential one. They were fitted using the ‘mgcv’ package in R version 3.6.

Step three, using the functional form of the relationship thus identified, the nonlinear model was re-run in SAS (PROC NLMIXED in SAS version 9.4) but using time as well as functions of temperature, solar radiation, RH and wind speed as suggested by visual examination of the GAM fits (Eq. 2). The fits of the model were plotted against time for each predictor in turn, holding the others constant. Below, we describe the method in more detail. The two PCS (‘VOEN’ and ‘NETTED’) were analysed separately.

Looking at the GAM plots it was determined that there were quadratic trends with temperature, solar radiation, and wind speed, but for RH a segmented model was used which had a linear slope for RH ≤ 60, and a different slope for RH > 60.

The final nonlinear model that included time and the functional relationships identified from the GAMs was fitted using PROC MIXED in SAS. It was of the form:

$$SF=\left(BT+CS+RHe+Ws+{Br}_{day}\right)\times exp\left(\frac{-{\left(Halhour-M\right)}^{2}}{S}\right),$$

in which

$$BT=BT0+BT1 \times temperature+BT2 \times temperatur{e}^{2},$$

$${CS=C1 \times solar+C2 \times solar}^{2},$$

$${W}_{s}={Ws}_{1}\times windspeed+{Ws}_{2}\times {windspeed}^{2},$$

and

$${RH}_{e}=\left\{\begin{array}{c}R1\times RH,if \, RH\le 60\\ R2\times RH,if \, RH>60\end{array}\right..$$

There is also a normal random effect for each day:

$${Br}_{day} \sim N \left(0, sB\right).$$

Last the model assumes that the predicted values are normally distributed:

2 $$sapflow \sim N \left(SF, S2\right).$$

The meaning of the terms in Eq. (2) are given in Abbreviations.

## Results

Given there was only one weather station per PCS this section is a descriptive outline of the climate data only with no formal statistical analysis possible. The contrast in average daily temperature tended to increase as the season progressed with temperatures under the rain covered PCS consistently tending higher than those under netted PCS (Table 1). Elevated temperatures under rain covered PCS resulted in an increased calculation of 86 growing degree days (GDD) compared to the netted PCS at time of harvest. Average minimum RH under rain covered and netted PCS were 55.4% and 51.9% respectively. Therefore, minimum RH under rain covered tended to be on average 6.5% greater than minimum RH measured under netted PCS. Solar radiation and wind speeds consistently tended to be lower under rain covered compared to netted PCS. The largest difference of all variables measured were wind speeds which tended to be lower (approximately three to fivefold) under rain covered PCS.

**Table 1:** Average climate data over the season for both rain covered (RC) and netted (NET) blocks.

	Temp (Celsius)		Max temp (Celsius)		Ave RH (%)		Min RH (%)		Solar radiation (MJ/m^2^/day)		Ave wind speed (m/s)		Ave VPD (kPa)		ETo (mm/day)	
	RC	NET	RC	NET	RC	NET	RC	NET	RC	NET	RC	NET	RC	NET	RC	NET
Nov	12.9	12.3	20.6	19.2	73	72.5	52.6	49.2	14.7	15.7	0.2	1.1	1.11	1.11	2.6	2.8
Dec	14.9	14.2	23.5	22.2	74.7	72.9	54.0	48.7	15.5	18.3	0.3	1.1	1.39	1.53	2.9	3.5
Jan	16.9	16.2	25.6	24.1	73	73.2	52.0	48.6	13.9	14.6	0.3	0.9	1.67	1.67	2.8	3.3
Feb	15.1	14.1	23.6	21.1	78.3	79.8	57.9	56.4	11.7	11.9	0.2	0.6	1.22	1.12	2.2	2.5
Mar	13.5	12.8	21.2	20.0	81.4	82.8	60.4	56.8	8.6	9.2	0.2	0.7	1.01	1.06	1.6	1.9

*MJ* mega joules, *VPD* vapor pressure deficit, *ETo* evapotranspiration.

**Figure 1:**
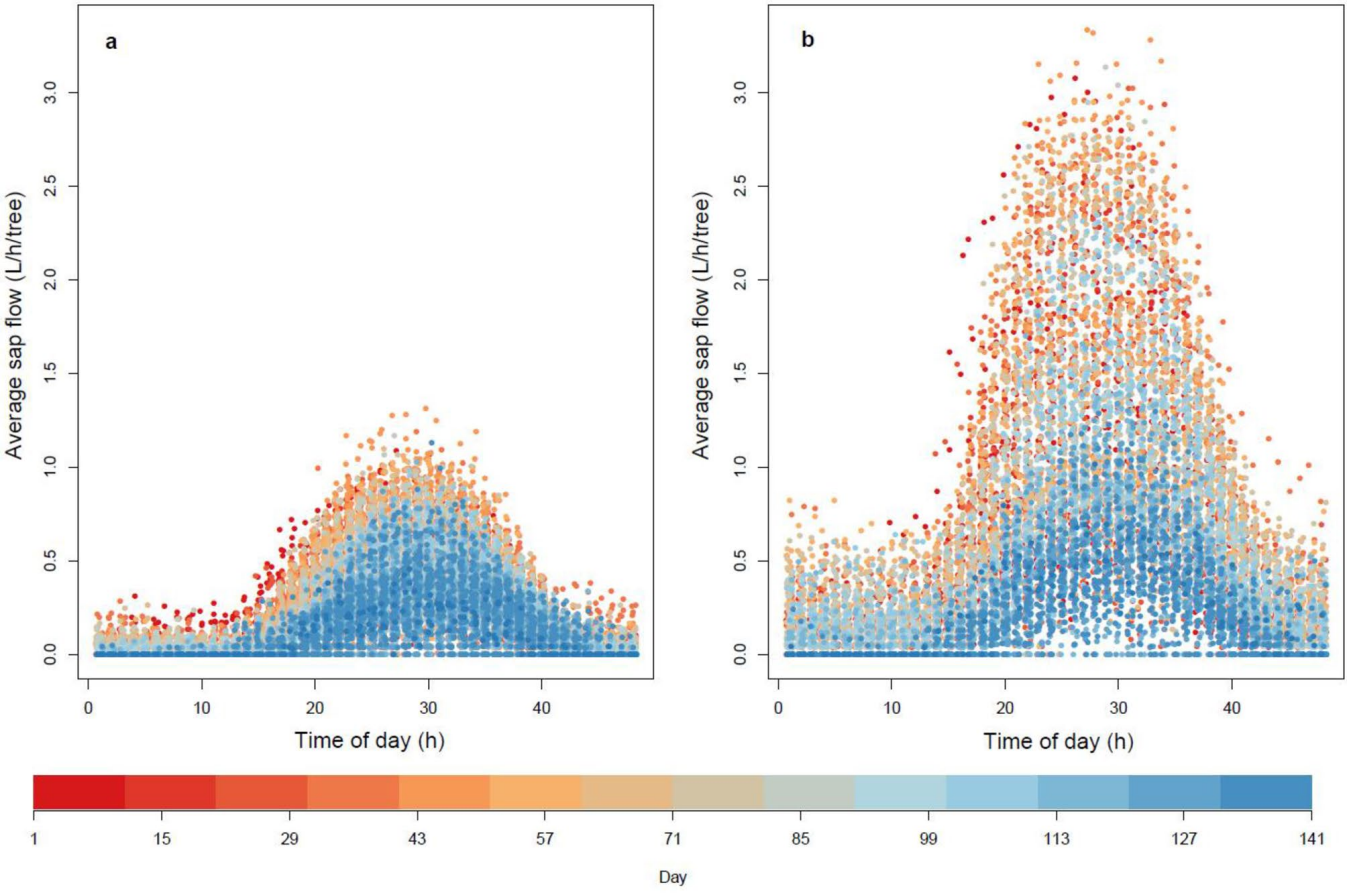
Observed average daily sap flow data for trees in (**a**) rain covered and (**b**) netted orchard blocks throughout the season. Average sap flow (L/h/tree) calculated from the sum of all sap flux velocities at each trunk depth (5, 15, 25 and 40 mm) using the respective sapwood area (cm^2^). Day 1, on November 11th, 2019, was when the rain covers were installed.

As shown in Fig. 1, the daily variation in sap flow followed a bell-shaped curve with a peak at approximately 2 pm. There was considerable variation between days within the season. Higher sap flows for both blocks were evident early in the season (days 1–71). Maximum sap flows were generally approximately threefold higher in netted than rain-covered trees. Average trunk circumferences in the netted block (48 cm) were 20% larger than those in rain covered block (40 cm).

**Figure 2:**
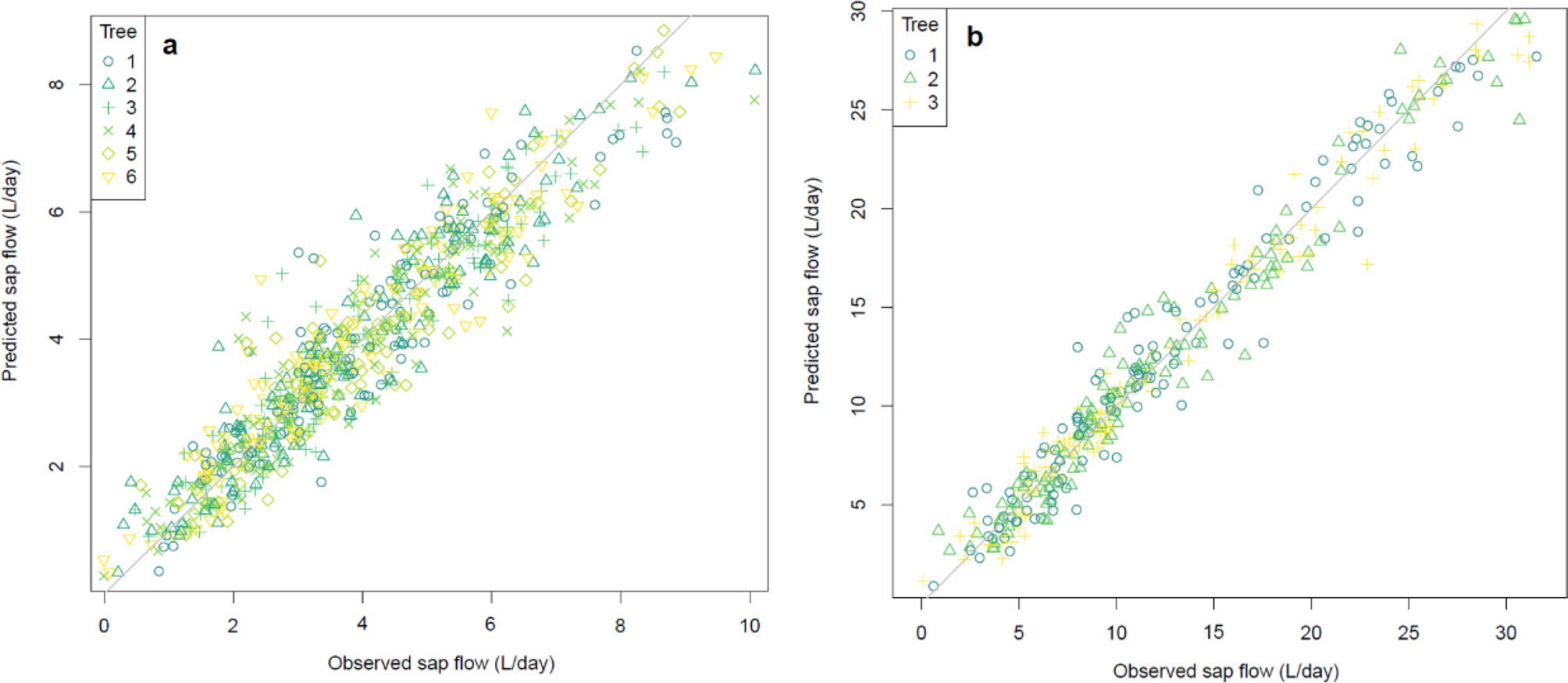
Predicted versus observed sap flow per tree for the entire growing season from 11/11/2019 to 31/3/2020 under (**a**) rain covered and (**b**) netted PCS. The line of equality is shown as a diagonal line. The r-square for the overall fit is 88.05% and 95.39% for rain covered and netted respectively.

Using only time, the predicted daily sap flow versus observed using Eq. (1) achieved an overall fit of 88% and 95% for rain covered and netted PCS, respectively (Fig. 2a,b). The model tended to overestimate daily sap flow readings on high sap flow days (> 6 L/day for rain covered and > 15 L/day for netted: results not shown). There were no outlying data points recorded during the season.

**Figure 3:**
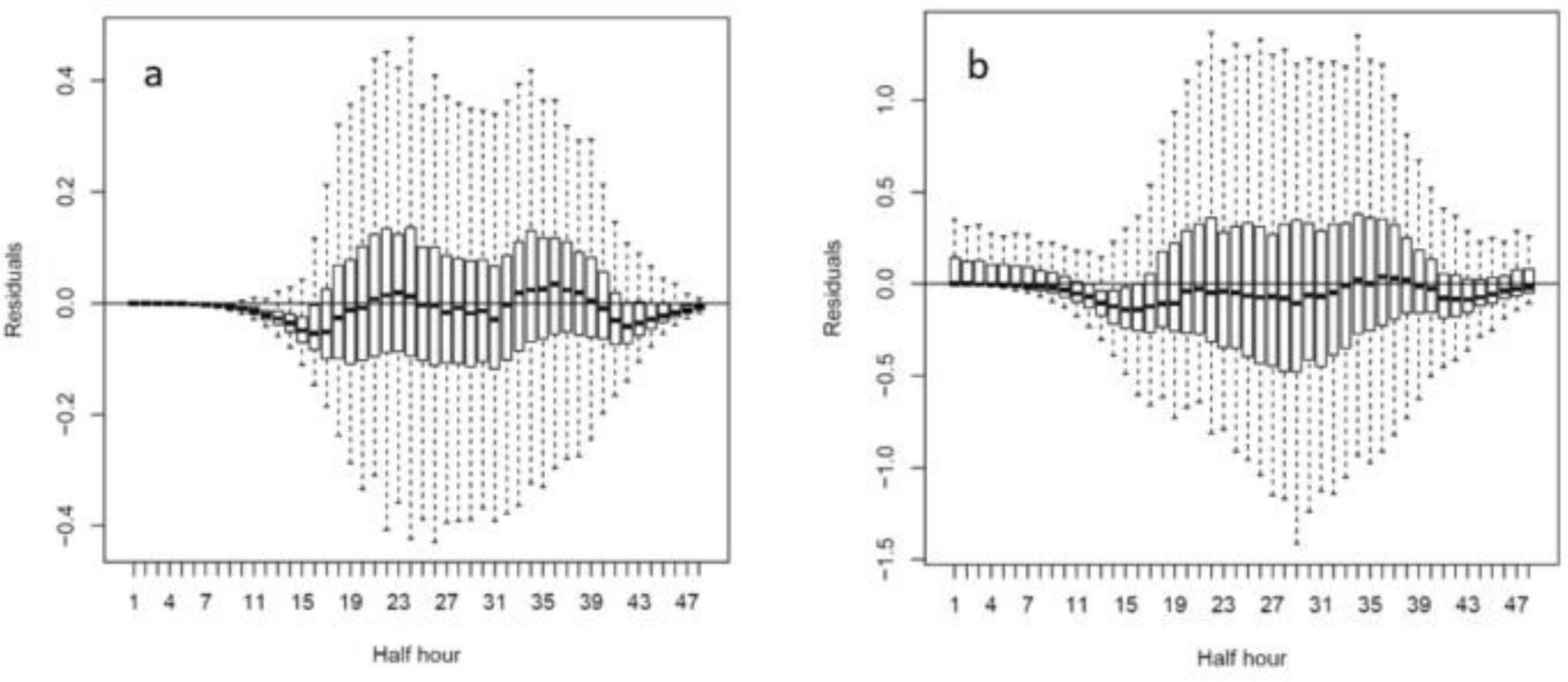
Boxplots of (**a**) rain covered and (**b**) netted showing the residuals per 30-min from the time-only model (Eq. 1).

Prediction of sap flow by time was significant (Eq. 1) (p < 0.0001). However, there were discrepancies in the residuals at approximately 15 and 42 half-hours (Fig. 3a,b) that warranted further investigation.

### GAM models

GAM plots illustated the residuals found in Fig. 3 (Eq. 1) and showed that the trends for each variable were complex. They indicated the source of variation in sap flow when time by itself could not. To fit an overall model it was necessary to simplify the trends. Accordingly, quadratic trends were assumed for temperature, solar and wind speed. For RH a segmented model, two regressions with a break point at RH = 60 (no lag), was assumed after analysis for the possiblity of a time lag effect using time lag plots (Table 2). The lack of a negative difference between the base model (lag 0, break point 60) with alternative models indicates that the original model is the best fit for the data.

**Table 2:** Differences in the AIC statistics relative to the base model (lag 0, break point 60).

Obs	diff1_60	diff2_60	diff3_60	diff1_40	diff0_40	diff2_40	diff3_40
1	1217.22	2193.21	3111.99	1802.06	886.271	2453.6	3202.65

Column names starting with diff1 indicates lag 1 (30 min), diff2 indicates lag 2 (60 min), diff3 indicates lag 3 (90 min). Column names ending in 40 indicate a RH break point of 40, whereas those ending in 60 indicate a RH break point of 60.

**Figure 4:**
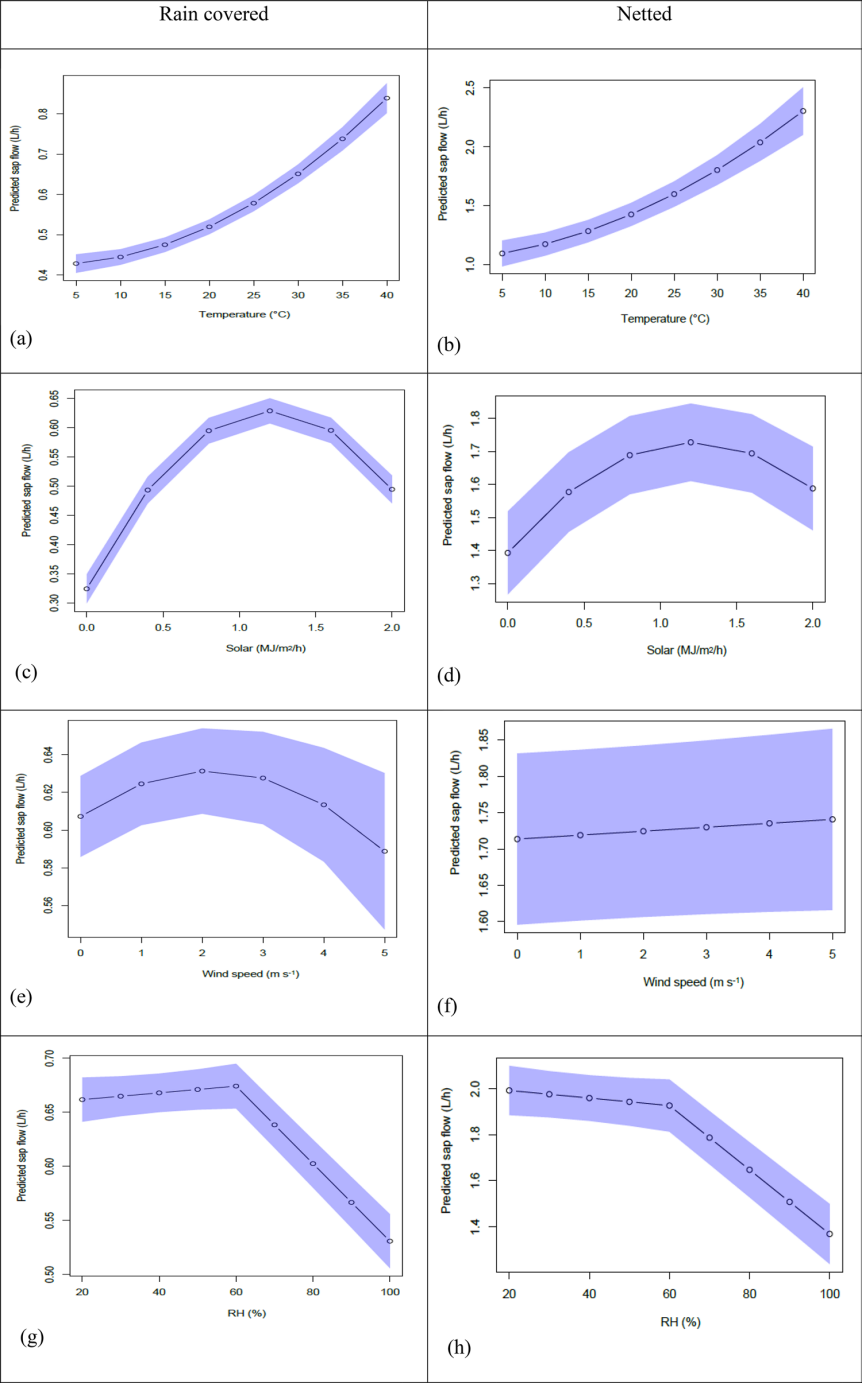
Fits from the statistical model of sap flow vs each predictor of sap flow in trees under either rain covered or netted PCS (separate scaling) with 95% confidence intervals shown in blue. (**a,b**) Temperature, (**c,d**) solar radiation, (**e,f**) wind speed, (**g,h**) relative humidity.

After assessing the GAM fits (Supplementary Figs. 1, 2) functional relationships were identified (Fig. 4) and were included in the full model involving time (Eq. 2). Some of the functional relationships included quadratic terms. The assumption of quadratic trends for temperature, solar, and wind speed were considered reasonable on the basis of published results^17,34^.

From the full model that included time and the GAM functional relationships, we plotted the predicted curves for each term holding others constant. Predicted sap flow increased exponentially with temperature to a maximum of 0.8 and 2.3 L per hour (L/h) at 40 °C for rain covered and netted PCS respectively. This prediction correlates with actual sap flows and temperature data recorded on the 31st of January 2020 (data not shown). Under rain covered PCS, predicted sap flow of 0.63 L peaked at a solar radiation of 1.2 MJ/m^2^/h before decreasing with a similar pattern under netted with a peak of 1.7 L at 1.2 MJ/m^2^/h albeit with greater variability. Predicted sap flow curvilinearly increased to 0.63 L/h at winds speeds of 2 m/s for rain covered however no effect was observed in netted. Predicted sap flow slightly increased at 20–60% RH for rain covered but in contrast linearly declined from 2.0 to 1.85 L/h between 20 and 60% RH for netted trees. At 60% RH sap flow inflected and linearly declined at a much greater rate up to 100% RH in trees of both treatments. Generally, variation was less for predictions of trees under rain covered than those for netted PCS.

## Discussion

A modelling approach was used to estimate the influence of climatic parameters on sap flow instead of the Penman–Monteith equation because the GAM ‘digressions’ allowed investigation of distinct climate parameters independent of the daily effects. The approach allowed the model to be constructed based on the data rather than any ‘forcing’ due to theoretical assumptions. The advantage of the GAMs was to identify if, once time was removed, the influence of these predictors on sap flow could be modelled as polynomials or other functions.

### Effects of protected cropping on mesoclimate

There was stark contrast in the mesoclimates of the rain covered relative to the netted block over the course of the growing season (Table 1). Deployment of the rain covers in early November resulted in consistently warmer temperatures, reduced light levels, and greatly reduced wind speeds relative to the netted PCS block. These are intuitive findings that are consistent with other reports^35–39^ although the differences were relatively minor in light and temperature and this is the first report of the effects on wind under rain covers to the best of our knowledge. In contrast, average and minimum RH, VPD and ETo were similar, although it is notable that average maximum VPD was only markedly lower under the covered PCS in December and only noticeably higher in February. This was unexpected given the consistently elevated temperatures under the rain covers and reflects the influence of RH. Therefore, even though the rain covered PCS was warmer, it was also more humid (higher minimum RH) which maintained VPD levels close to that under netted PCS in November, January, and March. In December, average maximum VPD under rain covers was lower (1.39 kPa) even though temperatures were on average 0.7 °C warmer. However, minimum RH was on average 10.3% lower under net counteracting the effect of increased temperatures under rain covers on VPD. In contrast Blanco et al.^39^ reported higher minimum RH levels and temperatures resulting in a 20% increase in VPD values at the beginning of the season for cherries under high tunnels compared to trees in the open. The 5–10 °C higher temperatures under high tunnels underpinned the 20% higher VPD and indicates that the relatively similar temperatures between rain covered and netted that we found may explain the relatively similar VPD values. In February, average maximum VPD values were 0.1 kPa higher under rain covers, whereby an average 1.0 °C higher temperature was more influential than the relatively small 2.6% difference in minimum RH. These results demonstrate the overriding influence of minimum, and not average, RH over temperature on VPD within rain covered PCS of a typical cool climate horticultural region where the rain covers are vented, on a slight slope and only continuous for approximately 150 m.

### Estimated sap flow responses to climate variables

#### Temperature and RH

The overall influence of modified climate in the rain covered PCS was manifest in the approximately threefold lower maximum sap flows, relative to netted, although the daytime bell-shaped pattern of sap flow was similar between trees of both rain covered and netted PCS (Fig. 1). Modelling sap flow using time as the main variable yielded r^2^ fits of 0.88 and 0.95 for rain covered and netted trees, respectively (Fig. 2a,b). Investigation of the residuals revealed under estimation of sap flow at approximately mid-morning and mid-afternoon (Fig. 3a,b). Thus, we took a GAMS approach, where all climatic variables bar one was held constant allowing investigation of how predicted sap flow varied with the individual climatic parameter of interest.

Of the variables investigated, temperature was associated with the highest sap flows under both rain covered and netted PCS (Fig. 4a,b). Predictions of temperature effects on sap flow showed an exponential trend with rates rapidly increasing at temperatures above 15 °C and maximum predicted sap flow coinciding with maximum daily temperatures of 39.1 °C and 38.2 °C measured for rain covered and netted PCS blocks, respectively. In the absence of literature to provide context to this novel finding, this may be contrary to reports of a parabolic relationship between temperature and photosynthetic rate in sweet cherry^40^ although we acknowledge the distinct influences of temperature on sap flow versus photosynthesis and further the major distinctions between our study and the rootstock, variety and growing environment of that reported by Sams and Flore^40^. Our study shows that a high temperature threshold for sap flow of sweet cherry under orchard conditions has not been established where the trees are acclimated to high temperatures^41^ and soil water is not limiting.

Stomatal closure has been noted at VPDs of > 1.5 kPa in potted sour cherry grown within a shade house where soil moisture was not limiting^42^. Although not directly measured we do not believe stomatal closure was evident in our study within an orchard setting. Daily average maximum VPDs under rain covers and netting of 1.7 kPa were associated with maximum daily sap flow at air temperatures close to 40 °C and very low RH. Stomatal closure may not have occurred when VPD’s were > 1.5 kPa in our study due to acclimation of leaves to higher light, temperatures and VPDs, relative to the sour cherry trees that were raised in pots in a shade house reported by Beckman et al.^42^. Urban et al.^19^ found that when VPDs were raised in a controlled environment, the effect of temperature on stomatal conductance was disproportionally larger than when VPD was low.

The investigation of a possible time lag in relationship between sap flow and RH driven by stem-storage of water found a possible break point at a RH of 40% in the segmented model using time lags of 1, 2 and 3 in comparison to the RH of 60% for the 0 (no lag) model. However we found that none of the time lag models (1, 2 and 3), compared using AIC^33^; better explained the data than the base model of no lag which indicates that stored water does not discernibly affect early morning sap flow in sweet cherry. Sap flow rates were not influenced by RH levels between 20 and 60% under rain covered PCS and declined only slightly between 20 and 60% RH under netted PCS. Low RH levels are generally associated with warm, dry conditions and the presence of wind which has a significant impact on the thickness of the boundary layer surrounding the leaf that consequently enables high rates of transpiration and therefore increased sap flow^43^. A critical threshold point was found whereby at approximately 60% RH, sap flows dramatically reduced in trees under both rain covered and netted PCS with increasing RH (Fig. 4g,h), presumably due to the decrease in driving force of transpiration and increasing effect of the leaf boundary layer. Similarly, Juhász et al.^44^ reported that vapor pressure, but not air temperature, was a major factor determining sap flow of field grown sweet cherries, although the lack of temperature influence may have been due to the relatively small sampling period of 45 days across the growing season compared to the 142 days measured in this study. A limitation of the GAMS approach is the prediction of approximately 0.53 L/h sap flow at 100% RH which is likely due to limited raw data at very high RH included in the model. Some of this apparent sap flow could be speculated to be due to refilling of internally stored water, although minimal apparent night-time sap flow indicates this to be at a very low level and the focus of this paper is day-time sap flow given its importance to fruit growth and quality. Overall, we highlight the 60% RH critical threshold as a novel contribution to the literature as we are not aware of any study investigating this critical relationship.

In summation, our findings indicate that under conditions of adequate water availability, high sap flows in ‘Lapins’ sweet cherry grafted to ‘Colt’ at high temperatures and low RH are likely enabled by high stomatal conductance. Conversely RH > 60% (and lower VPD) is associated with rapidly decreasing sap flows that we speculate are due to an increase in the thickness of leaf boundary layers, that limit leaf gas exchange.

#### Wind

Despite large confidence intervals relative to the other parameters investigated, sap flow was associated with wind in a parabolic response for trees under rain covered PCS with a maximum sap flow occurring at approximately 2 m/s with little to no response in trees under netted PCS. Overall fluctuation in sap flow quantity did not vary greatly with increasing wind speeds, although the parabolic response under rain covered PCS is notable. Low wind speeds are associated with high RH^45,46^ which could explain the curvilinear increase of sap flow from 0 up to approximately 2 m/s. Average wind speeds under the rain covers were considerably lower (0.2 m/s) in contrast to under netted (0.9 m/s), therefore the GAM response curve illustrating sap flow declining under rain covers when wind speed are > 2 m/s more than likely rarely occurred. If wind speeds did reach > 2 m/s a reduction in sap flow could be due to effects of wind on the closure of stomata in response to increased rates of evapotranspiration^47^. Unfortunately, measurement of leaf conductance was outside the scope for this study at the time yet warrants further investigation. Linear trends of transpiration would be expected for leaves with relatively high rates of stomatal conductance in response to increasing wind velocity up to a critical threshold, while a saturated response to an increase in wind speeds would be expected for leaves with relatively low rates of stomatal conductance^17^. Results under rain covered PCS are consistent with stomata that are responsive to water loss under conditions of high wind. Intuitively we expect wind to have a greater influence on the transpiration and sap flow of trees under rain covers due to the reduction of humidity levels that can develop during periods of little or no wind. This may explain why we observed a parabolic response under the rain covers in contrast to the lack of response under netted where trees are directly impacted by wind effects.

Overall, the highest modelled sap flow under rain covered PCS occurred when tree canopies were exposed to moderate wind speeds of 2 m/s. Higher wind speeds would displace the rain cover vents which would lower RH under the rain covers, reducing the boundary layer surrounding the leaf, creating a higher VPD and resulting in an increase in transpiration and subsequent sap flow rates. While average wind speeds of 1 m/s under netted would continually displace any build up of RH in the canopy resulting in a reduction in boundary layer thickness subsequently leading to higher levels of transpiration and sap flow.

#### Light interception

The strong correlation found between increased global radiation levels and tree sap flow rates in all trees measured (Fig. 4c,d) is supported by Juhász et al.^48^ who reported that global radiation was the driving force of sap flow when light intensities reached levels > 200 W/m^2^ in sweet cherries. Further, Green et al.^28^ found that global short wave radiation and VPD were the main driving forces of sap flow in 1-year old *Malus* × *domestica* (Envy) apples. Sap flow in trees under rain covered and netted PCS peaked at approximately 1.2 MJ/m^2^/h before declining with any further increase in solar radiation. Interestingly the 95% confidence intervals were tighter under the rain covered in contrast to under the netted block, however this may be due to the larger number of trees studied under rain covered PCS. Overall, these results are consistent with stomatal closure at maximum light levels resulting in a reduction in photosynthesis as widely reported^49,50^ though this is difficult to reconcile with the lack of similar response to high temperatures.

Underestimation of modelled daily sap flow at 15 and 42 half hours (7am and 9 pm) (Fig. 3), may be due to light interception by the ‘walls’ of the tree canopy due to the solar zenith angle of between 70° and 80° across the measurement period that was not accurately captured by the horizontally oriented light quantum sensors of the weather stations. Higher actual, than estimated, leaf light interception lower in the canopy may be a cause for the higher sap flow observed at morning and evening than what the model predicted based on light estimates of the horizontally oriented light sensors.

## Conclusion

This research has demonstrated the overriding influence of minimum, and not average, RH on tree sap flow under PCS. Further, the study has discovered that whilst time alone can predict sap flow, a modelling approach can highlight the influences of individual climatic parameters on sap flow, in this case in the context of ‘Lapins’ scion on ‘Colt’ rootstock (acknowledging that results are influenced by scion and rootstock interactions). The distinct mesoclimates between the PCS were reflected in different sap flow responses to humidity and wind driven by acclimation of trees. Sap flow response was similarly affected by light and temperature, probably due to effects on VPD and stomatal response. Overall, the critical threshold response of sap flows greater than 60% RH is a notable finding of how tree canopies respond to climatic variables and may be the major driver underpinning the very significantly lower (threefold) maximum sap flow in rain covered, relative to netted, PCS. Although this could indicate that productivity might be lower under covers due to CO_2_ limitation to photosynthesis, the opposite is observed (anecdotally) in terms of tree canopy development and fruit yield. Thus, the consistently warmer temperatures and GDD presumably drive greater canopy level photosynthesis and lower average wind speeds are yet adequate to prevent RH-induced limitation to stomatal conductance and CO_2_ uptake. We have discounted the possibility that stem-stored water might induce a lag in the relationship between sap flow and RH during the early morning hours and we acknowledge that night-time stem re-filling might contribute to the apparent night-time tree sap flow between the two blocks. However this was not a focus of the study whereas the 200% decrease in daily water uptake in trees under rain covers relative to those under netting and how this was affected by various climate parameters were key findings in relation to the objectives of the study. From a practical perspective, the application of ETo calculations with regard to irrigation and fertigation scheduling under rain covered PCS is of interest. Although daily ETo levels correlated strongly with tree water use (Stone unpublished), the relationship between ETo and tree water use varied between the netted and rain covered PCS. However, the use of GAMs to further inform our understanding of each climatic predictor and its effect on tree sap flow is invaluable for understanding tree water-use physiology in response to climate and will lead to the improvement of efficiency in irrigation and fertigation applications. The reduced overall tree water use under rain covers measured in this study, the influence of RH and VPD, and indirectly of wind, and the potential long-term effects on nutrient uptake and partitioning, and subsequent fruit quality along with the effect soil moisture requires further research.

## Supplementary Information

Supplementary Information.

## Data Availability

The datasets generated during and/or analysed during the current study are available due to the vast quality of raw data collected throughout the trial, however will be available from the corresponding author on reasonable request.

## Abbreviations

SF: Sap flow
B: Mean estimate for the maximum sap flow
M: Mean half hour when the sap flow is maximum
S: Variability for the time when sap flow was maximum
BT0: Regression intercept
BT1: Linear effect for temperature
BT2: Quadratic effect for temperature
C1: Linear effect for solar
C2: Quadratic effect for solar
Ws1: Linear effect for wind speed
Ws2: Quadratic effect for wind speed
R1: Constant effect for RH ≤ 60
R2: Linear effect for RH > 60
sb: Variance for the random tree effect
S2: Variance for the prediction

## References

[1] Jensen MH, Malter AJ (1995). Protected Agriculture—A Global Review. World Bank Technical Paper Number 253.

[2] Meli T, Riesen W, Widmer A (1984). Protection of sweet cherry hedgerows with polyethylene films. Acta Hortic..

[3] Janick J (2004). Horticultural Reviews.

[4] Janke RR, Altamimi ME, Khan M (2017). The use of high tunnels to produce fruit and vegetable crops in North America. Agric. Sci..

[5] Alarcon JJ (2000). Sap flow as an indicator of transpiration and the water status of young apricot trees. Plant Soil.

[6] Ferrara G, Flore J (2003). Comparison between different methods for measuring tranpiration in potted apple trees. Biol. Plant..

[7] Nicolás E, Torrecillas A, Amico JD, Alarcón JJ (2005). Sap flow, gas exchange, and hydraulic conductance of young apricot trees growing under a shading net and different water supplies. J. Plant Physiol..

[8] Green S, Romero R (2012). Can we improve heat-pulse to measure low and reverse flows. Acta Hortic..

[9] Noitsakis B, Nastis AS (1995). Seasonal changes of water potential, stomatal conductance and transpiration in the leaf of cherry trees grown in shelter. CIHEAM.

[10] Lang GA (2009). High tunnel tree fruit production: The final frontier. HortTechnology.

[11] Lang GA (2013). Tree fruit production in high tunnels: Current status and case study of sweet cherries. Acta Hortic..

[12] Meland M, Frøynes O, Kaiser C (2017). High tunnel production systems improve yields and fruit size of sweet cherry. Acta Hortic..

[13] Cohen S, Moreshet S, Guillou LL, Simon J-C, Cohen M (1997). Response of citrus trees to modified radiation regime in semi-arid conditions. J. Exp. Bot..

[14] Zeppel M, Murray BR, Barton C, Eamus D (2004). Seasonal responses of xylem sap velocity to VPD and solar radiation during drought in a stand of native trees in temperate Australia. Funct. Plant Biol..

[15] Bonada M, Buesa I, Moran MA, Sadras VO (2018). Interactive effects of warming and water deficit on Shiraz vine transpiration in the Barossa Valley, Australia. OENO One.

[16] Wang KY, Kellomaki S, Zha T, Peltola H (2005). Annual and seasonal variation of sap flow and conductance of pine trees grown in elevated carbon dioxide and temperature. J. Exp. Bot..

[17] Laplace S, Chu C, Kume S (2013). Wind speed response of sap flow in five subtropical trees based on wind tunnel experiments. Br. J. Environ. Clim. Change.

[18] Kellomäki S, Wang KY (2002). Sap flow in Scots pine growing under conditions of year-round carbon dioxide enrichment and temperature elevation. Plant, Cell Environ..

[19] Urban J, Ingwers M, McGuire MA, Teskey RO (2017). Stomatal conductance increases with rising temperature. Plant Signal. Behav..

[20] Wu J (2020). Nocturnal sap flow is mainly caused by stem refilling rather than nocturnal transpiration for *Acer truncatum* in urban environment. Urban For. Urban Green..

[21] Chen Y-J (2015). Time lags between crown and basal sap flows in tropical lianas and co-occurring trees. Tree Physiol..

[22] Marshall DC (1958). Measurment of sap flow in conifers by heat transport. Plant Physiol..

[23] Swanson RH, Whitfield WA (1981). A numerical analysis of heat pulse velocity theory and practice. J. Exp. Bot..

[24] Green S, Clothier B, Jardine B (2003). Theory and practical application of heat pulse to measure sap flow. Am. Soc. Agron..

[25] Goodwin I, Cornwall D, Green SR (2012). Pear transpiration and basal crop coefficients estimated by sap flow. Acta Hortic..

[26] Fernandez JE (2001). Heat-pulse measurements of sap flow in olives for automating irrigation, tests, root flow and diagnostics of water stress. Agric. Water Manag..

[27] Green SR, Clothier B (1988). Water use of kiwifruit vines and apple trees by the heat-pulse technique. J. Exp. Bot..

[28] Green SR (2018). Measurement of sap flow in young apple trees using the average gradient heat-pulse method. Acta Hortic..

[29] Green S, Clothier B, Perie E (2009). A re-analysis of heat pulse theory across a wide range of sap flows. Acta Hortic..

[30] Allen RG, Pereira LS, Raes D, Smith M (1998). Crop Evapotranspiration Guidelines for Computing Crop Water Requirements, FAO Irrigation and Drainage Paper 56.

[31] *R: A Language and Environment for Statistical Computing* (R Foundation for Statistical Computing, 2010).

[32] Hastie T, Tibshirani R (1990). Generalized Additive Models.

[33] Akaike H (1974). A new look at the statistical model identification. IEEE Trans. Autom. Control.

[34] Sams CE, Flore JA (1982). The influence of leaf age, leaf position on the shoot, and environmental variables on net photosynthetic rate of sour cherry (*Prunus cerasus* L. 'Montmorency'). J. Am. Soc. Hortic. Sci..

[35] Wallberg BN, Sagredo KX (2014). Vegetative and reproductive development of 'Lapins' sweet cherry trees under rain protective cropping. Int. Soc. Hortic. Sci..

[36] Lang GA (2014). Growing sweet cherries under plastic covers and tunnels: Physiological aspects and practical considerations. Acta Hortic..

[37] Goodwin I, McClymont L, Turpin S, Darbyshire R (2018). Effectiveness of netting in decreasing fruit surface temperature and sunburn damage of red-blushed pear. N. Z. J. Crop. Hortic. Sci..

[38] Mika A, Buler Z, Wójcik K, Konopacka D (2019). Influence of the plastic cover on the protection of sweet cherry fruit against cracking, on the microclimate under cover and fruit quality. J. Hortic. Res..

[39] Blanco V, Zoffoli JP, Ayala M (2019). High tunnel cultivation of sweet cherry (*Prunus avium* L.): Physiological and production variables. Sci. Hortic..

[40] Sams CE, Flore JA (1983). Net photosynthetic rate of sour cherry (*Prunus cerasus* L. ‘Montmorency’) during the growing season with particular reference to fruiting. Photosynth. Res..

[41] Lange OL, Schulze ED, Evenari M, Kappen L, Buschbom U (1974). The temperature-related photosynthesis capacity of plants under desert conditions. Oecologia.

[42] Beckman TG, Perry RL, Flore JA (1992). Short-term flooding affects gas exchange characteristics of containerized sour cherry trees. HortScience.

[43] Lei H, Zhi-Shan Z, Xin-Rong L (2010). Sap flow of Artemisia ordosica and the influence of environmental factors in a revegetated desert area: Tengger Desert, China. Hydrol. Processes.

[44] Juhász, A., Hrotko, K. & Tokei, L. *Air and Water Components of the Environment,* 76–82.

[45] Ravi S, D'Odorico P (2005). A field-scale analysis of the dependence of wind erosion threshold velocity on air humidity. Geophys. Res. Lett..

[46] Holmes, M. & Farrell, D. *South African Avocado Growers Association Yearbook* Vol. 16, 59–64 (1993).

[47] Jones HG (2014). Plants and Microclimate: A quantitative Approach to Environmental Plant Physiology.

[48] Juhász Á, Sepsi P, Nagy Z, Tőkei L, Hrotkó K (2013). Water consumption of sweet cherry trees estimated by sap flow measurement. Sci. Hortic..

[49] Gussakovsky EE, Salomon E, Ratner K, Shahak Y, Driesenaar ARJ (1993). Photoinhibition (light stress) in citrus leaves. Acta Hortic..

[50] Grappadelli LC, Lakso AN (2007). Is maximizing orchard light interception always the best choice?. Acta Hortic..

