# Supplementary material for: Sap flow of sweet cherry reveals distinct effects of humidity and wind under rain covered and netted protected cropping systems
Source: Sci Rep. 2022 Dec 5;12:21031. doi: 10.1038/s41598-022-25207-0 (PMC9722905; doi:10.1038/s41598-022-25207-0)
Supplement: Supplementary file 1 — Supplementary Information. [file 41598_2022_25207_MOESM1_ESM.pdf]

Supplementary Data

Supplementary Table 1: Parameter estimates for the time only model for rain covers

Parameter	Estimate	Standard Error	DF	t Value	Pr > |t|	95% Confidence Limits		Gradient
M	28.9222	0.02040	141	1417.80	<.0001	28.8819	28.9625	0.000025
S	95.1387	0.5287	141	179.96	<.0001	94.0936	96.1838	3.057E-7
B	0.4844	0.01436	141	33.73	<.0001	0.4560	0.5128	0.000172
S2	0.01122	0.000081	141	138.34	<.0001	0.01106	0.01138	0.003319
sB	0.02906	0.003470	141	8.37	<.0001	0.02220	0.03592	0.003518

Supplementary Table 2. Parameter estimates for the time only model for netted

Parameter	Estimate	Standard Error	DF	t Value	Pr > |t|	95% Confidence Limits		Gradient
M	28.6404	0.03915	141	731.57	<.0001	28.5630	28.7178	-0.00552
S	123.94	1.2060	141	102.77	<.0001	121.56	126.33	0.000029
B	1.2623	0.04147	141	30.44	<.0001	1.1803	1.3443	-0.00744
S2	0.1184	0.001241	141	95.39	<.0001	0.1159	0.1208	-0.04439
sB	0.2396	0.02882	141	8.31	<.0001	0.1826	0.2966	0.007711

Supplementary Table 3: Regression coefficients for time and GAM functional relationships due to climate in the rain covered block.

Parameter	Estimate	Standard Error	DF	t Value	Pr > |t|	95% Confidence Limits		Gradient
M	28.2985	0.06842	135	413.59	<.0001	28.1632	28.4338	0.000421
S	296.80	6.9471	135	42.72	<.0001	283.06	310.54	-0.40786
BT00	0.3519	0.01914	135	18.38	<.0001	0.3140	0.3898	-0.00317
BT1	-0.1041	0.1050	135	-0.99	0.3233	-0.3117	0.1035	-0.01123
BT2	3.0060	0.2570	135	11.70	<.0001	2.4977	3.5142	-0.00304
C1	0.5396	0.009099	135	59.31	<.0001	0.5216	0.5576	-0.00616
C2	-0.2245	0.004571	135	-49.12	<.0001	-0.2336	-0.2155	0.011654
R1	0.000332	0.000277	135	1.20	0.2315	-0.00021	0.000880	-0.16309
R2	-0.00382	0.000153	135	-24.93	<.0001	-0.00412	-0.00352	-1.20422
ws1	0.02399	0.003256	135	7.37	<.0001	0.01756	0.03043	-0.00190
ws2	-0.00559	0.001208	135	-4.62	<.0001	-0.00798	-0.00320	-0.10591
S2	0.008364	0.000062	135	134.76	<.0001	0.008241	0.008486	-5.76970
sb	0.002159	0.000279	135	7.75	<.0001	0.001608	0.002711	3.00381

Supplementary Table 4: Regression coefficients for time and GAM functional relationships due to climate in the netted block.

Parameter	Estimate	Standard Error	DF	t Value	Pr > |t|	95% Confidence Limits		Gradient
M	27.6225	0.09643	135	286.44	<.0001	27.4318	27.8132	-0.00333
S	266.51	7.5356	135	35.37	<.0001	251.61	281.41	7.867E-6
BT00	1.5349	0.09326	135	16.46	<.0001	1.3505	1.7193	0.005718
bt1	0.6814	0.5065	135	1.35	0.1808	-0.3204	1.6832	-0.00161
BT2	6.5470	1.3338	135	4.91	<.0001	3.9091	9.1849	-0.00066
C1	0.5788	0.03602	135	16.07	<.0001	0.5075	0.6500	-0.00420
C2	-0.2382	0.01927	135	-12.36	<.0001	-0.2763	-0.2001	-0.00461
R1	-0.00172	0.001278	135	-1.35	0.1795	-0.00425	0.000803	0.17324
R2	-0.01466	0.000690	135	-21.26	<.0001	-0.01603	-0.01330	0.93176
ws1	0.005198	0.01237	135	0.42	0.6749	-0.01926	0.02966	-0.04561
ws2	0.000144	0.003169	135	0.05	0.9638	-0.00612	0.006412	-0.15410
S2	0.1019	0.001099	135	92.79	<.0001	0.09977	0.1041	-0.00451
sb	0.05670	0.007322	135	7.74	<.0001	0.04222	0.07118	-0.02528

Non-significant terms dropped

Parameter	Estimate	Standard Error	DF	t Value	Pr > |t|	95% Confidence Limits		Gradient
M	27.6224	0.09641	135	286.52	<.0001	27.4317	27.8130	-0.00587
S	266.51	7.5212	135	35.43	<.0001	251.64	281.39	-0.00001
BT00	1.5342	0.09066	135	16.92	<.0001	1.3549	1.7135	0.003913
bt1	0.6815	0.4928	135	1.38	0.1689	-0.2931	1.6562	0.000311
BT2	6.5460	1.3034	135	5.02	<.0001	3.9684	9.1236	0.000053
C1	0.5788	0.03588	135	16.13	<.0001	0.5078	0.6498	0.025278
C2	-0.2382	0.01923	135	-12.39	<.0001	-0.2762	-0.2002	0.037458
R1	-0.00172	0.001276	135	-1.35	0.1790	-0.00425	0.000800	-0.15940
R2	-0.01466	0.000670	135	-21.87	<.0001	-0.01598	-0.01333	0.53328
ws1	0.005703	0.005427	135	1.05	0.2952	-0.00503	0.01644	-0.02100
S2	0.1019	0.001099	135	92.79	<.0001	0.09977	0.1041	0.002465
sb	0.05670	0.007322	135	7.74	<.0001	0.04222	0.07118	0.010492

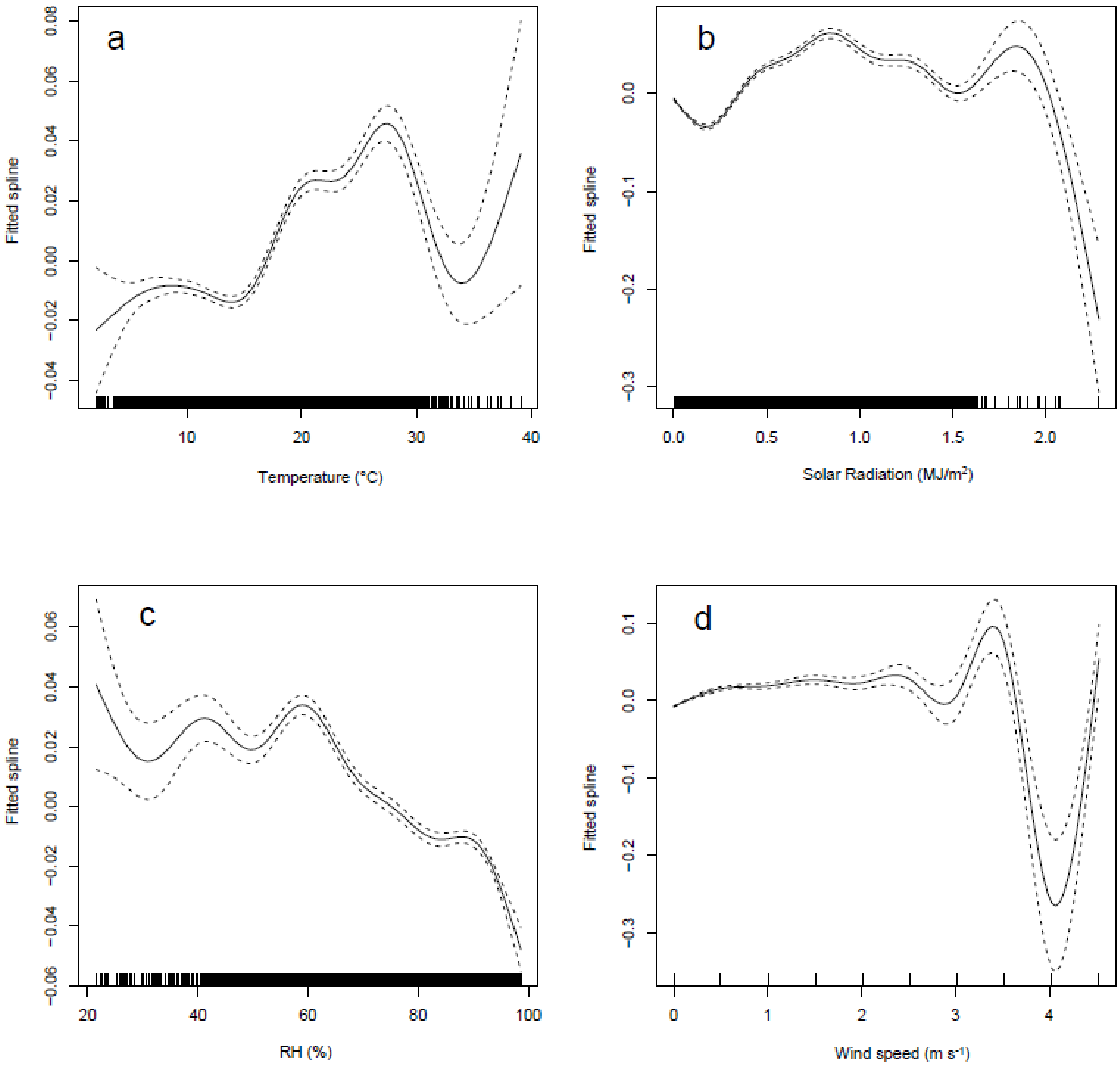

Supplementary Figure 1: Rain covered GAM models to the residuals of figure 4 with 95% confidence intervals.

(a) temperature, **(b)** solar radiation, **(c)** relative humidity, **(d)** wind speed.

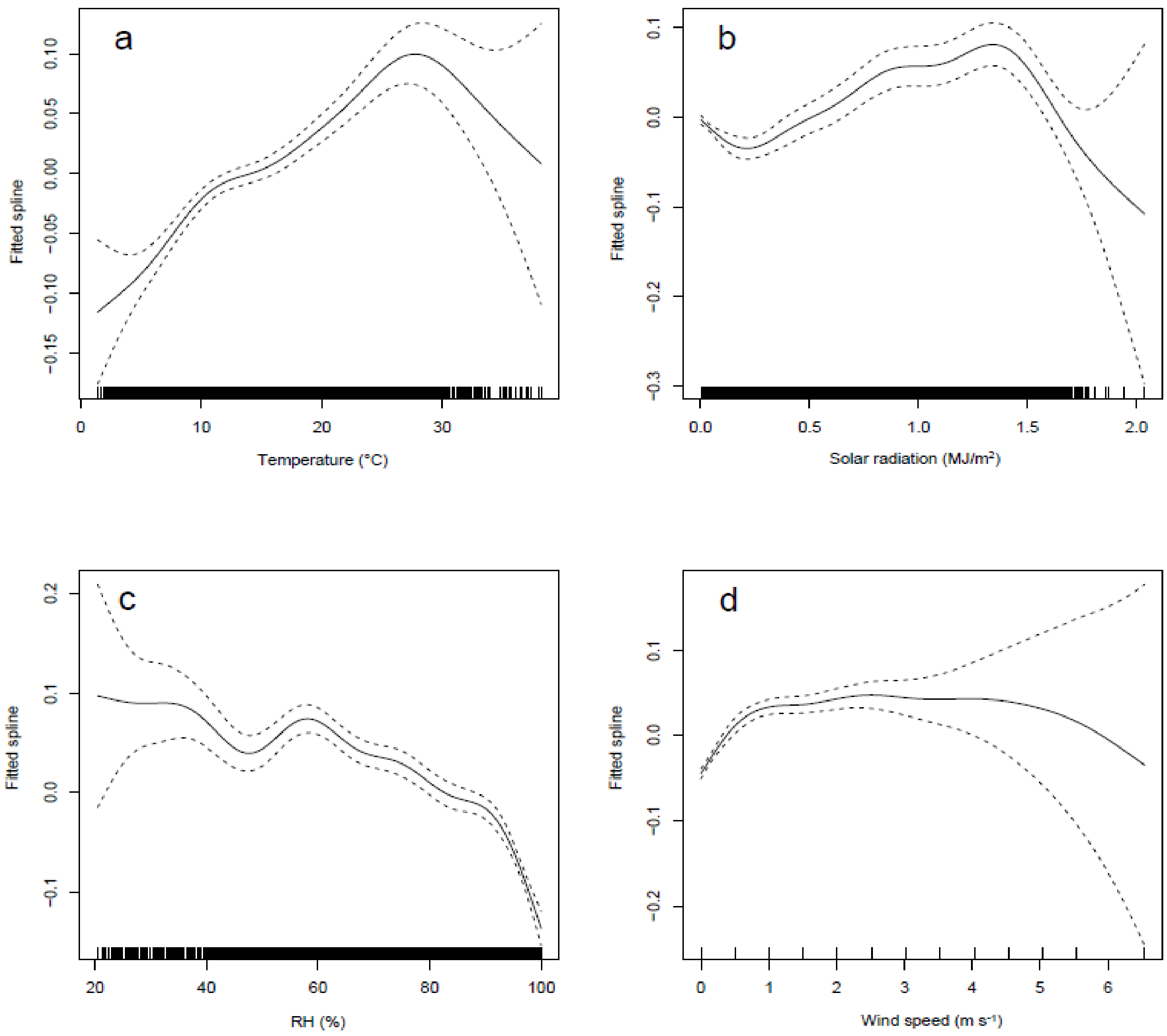

Supplementary Figure 2: Netted GAM models to the residuals of Figure 4 with 95% confidence intervals. **(a)** temperature, **(b)** solar radiation, **(c)** relative humidity, **(d)** wind speed

Model Prediction

The trends for each covariate are complex (justifying the GAMs approach). Values <0 indicate underestimation of sap flow by the initial nonlinear model using equation 1, while values >0 describe overestimation of sap flow. Trends for both blocks are similar indicating there not being a single factor uniquely associated with rain covered or netted that contributes to the residuals – instead, how the trees are responding to the environmental conditions at that time of day that is not ‘linearly predictable’.
